# Supplementary material for: Staphylococcus aureus Lung Infection Results in Down-Regulation of Surfactant Protein-A Mainly Caused by Pro-Inflammatory Macrophages
Source: Microorganisms. 2020 Apr 16;8(4):577. doi: 10.3390/microorganisms8040577 (PMC7232181; doi:10.3390/microorganisms8040577)
Supplement: Supplementary file 1 [file microorganisms-08-00577-s001.pdf]

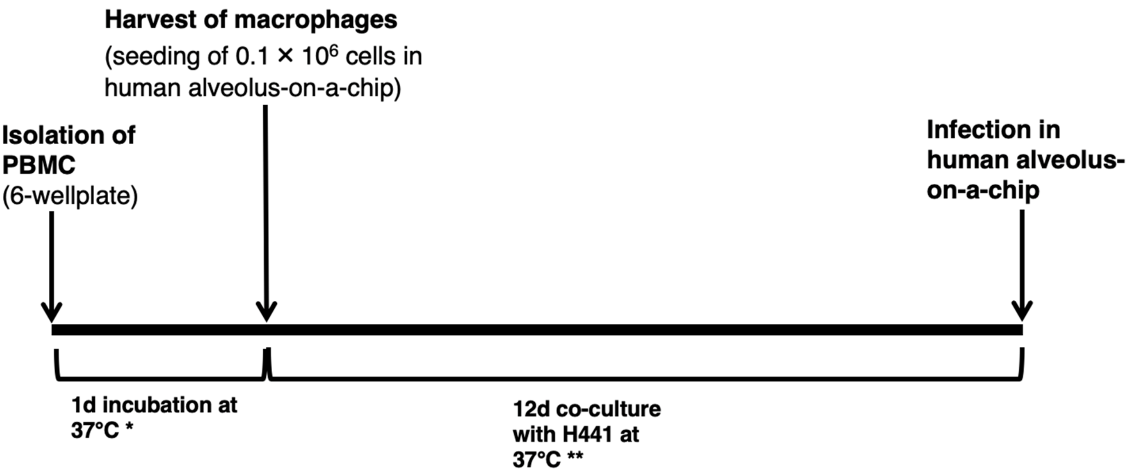

Figure S1. After the isolation of peripheral blood mononuclear cells (PBMCs) and the cultivation on 6-wellplates for one day with * RPMI (containing 10% autologous serum, 10 ng/ml M-CSF, 10 ng/ml GM-CSF and penicillin/streptomycin) the macrophages were harvested and seeded on the epithelial chamber with 0.1 x 0^6 cells in the human alveolus-on-a-chip model. After 12 days of co-culture with ** RPMI (containing 20% autologous serum, 10 ng/ml GM-CSF and dexamethason) the infection of the human alveolus-on-a-chip was performed.

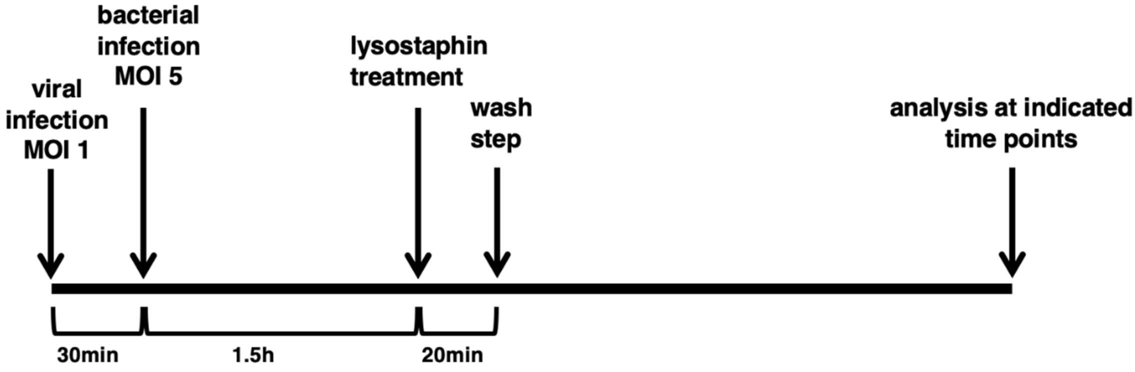

Figure S2. Overview of the infection procedure. The experiment begins with the viral infection of the cells by using the IV strain A/Puerto Rico/8 (MOI 1). Viral infection was conducted in RPMI (0.2% autologous human serum, 1 mM $MgCl_2$, 0.9 mM $CaCl_2$) and incubated for 30 min at 37 °C without centrifugation. The bacterial infection was performed with RPMI/BA (1% human serum albumin (HSA), 1mM 4-(2-hydroxyethyl)-1-piperazineethanesulfonic acid (HEPES)). After the bacterial infection, cells were incubated with bacteria for 90 min at 37 °C and 5% CO_2. Afterward, the cells were washed. To eliminate the non-internalized bacteria, cells were treated for 20 min with RPMI-medium containing lysostaphin at a concentration of 6 μg/mL for the NCI-H441 mono-cell culture and 20 μg/mL for the human alveolus-on-a-chip model. Subsequently, cells were washed with PBS and then incubated with RPMI for indicated time points at 37 °C with 5% CO_2. For the co-infection scenarios, both pathogens were added to the cells. For this, first, the infection with IV strain A/Puerto Rico/8 (MOI 1) was performed, followed by the bacterial infection with *S. aureus*/USA300/WT (MOI 5).

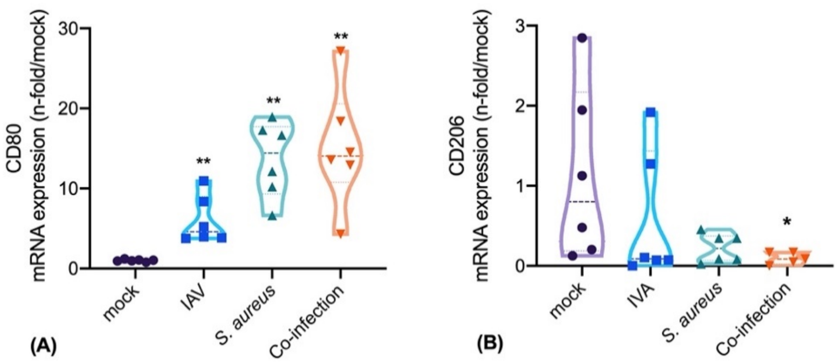

Figure S3. Human monocyte-derived macrophages (hMdM) were infected with IV A/Puerto Rico/8 (MOI 1), *S. aureus*/USA300/WT (MOI 5) or with both pathogens (co-infection). The mRNA expression of CD80 (A) was significantly higher after infection and the mRNA expression of CD206 (B) has been shown to be significantly lower within the co-infected cells. Mock-treated cells obtained the same medium and treatment as infected cells without the pathogen. Kruskal–Wallis and Mann–Whitney U test, * $p < 0.05$, ** $p < 0.005$.
